# The Production of Porous Hydroxyapatite Scaffolds with Graded Porosity by Sequential Freeze-Casting

**DOI:** 10.3390/ma10040367

**Published:** 2017-03-31

**Authors:** Hyun Lee, Tae-Sik Jang, Juha Song, Hyoun-Ee Kim, Hyun-Do Jung

**Affiliations:** 1 Department of Materials Science and Engineering, Seoul National University, Seoul 151-744, Korea; akusaya@snu.ac.kr (H.L.); cgamja@snu.ac.kr (T.-S.J.); kimhe@snu.ac.kr (H.-E.K.); 2 School of Chemical and Biomedical Engineering, Nanyang Technological University, 70 Nanyang Drive, Singapore 637457, Singapore; songjuha@ntu.edu.sg; 3 Biomedical Implant Convergence Research Center, Advanced Institutes of Convergence Technology, Suwon 443-270, Korea; 4 Liquid Processing & Casting Technology R&D Group, Korea Institute of Industrial Technology, Incheon 406-840, Korea

**Keywords:** freeze-casting, porous materials, porosity, graded structure, compressive strengths

## Abstract

Porous hydroxyapatite (HA) scaffolds with porosity-graded structures were fabricated by sequential freeze-casting. The pore structures, compressive strengths, and biocompatibilities of the fabricated porous HA scaffolds were evaluated. The porosities of the inner and outer layers of the graded HA scaffolds were controlled by adjusting the initial HA contents of the casting slurries. The interface between the dense and porous parts was compact and tightly adherent. The porosity and compressive strengths of the scaffold were controlled by the relative thicknesses of the dense/porous parts. In addition, the porous HA scaffolds showed good biocompatibility in terms of preosteoblast cell attachment and proliferation. The results suggest that porous HA scaffolds with load-bearing parts have potential as bone grafts in hard-tissue engineering.

## 1. Introduction

Series of calcium phosphate (CaP) materials have been extensively used in the field of orthopedic and dental implants because of their outstanding mechanical properties, corrosion resistance, and biocompatibility [1,2,3,4,5,6]. Among them, hydroxyapatite (HA, Ca_10_(PO_4_)_6_(OH)_2_) is one of the main components of bone and has the ability to promote osteoconduction [7]. Therefore, HA has been used in several ways, including as a coating material for enhancing the biocompatibility of pure materials [7,8,9,10,11], bone cement to compensate for bone loss during orthopedic surgery [12,13,14,15], and artificial bone scaffolds to replace autografts or allografts, which have limited donor availability [16,17]. However, dense HA has a low resorption rate under physiological conditions, which can result in poor fixation between the scaffold and the newly generated bone [7].

Porous bioceramics have been heavily researched and developed to overcome the abovementioned problems. Porous ceramics have pores that provide rough surfaces and are preferred by cells as the bone grows into the interconnected pores, improving the adhesion between the bone and implant. Furthermore, desirable properties for bone scaffolds can be obtained by regulating pore size and porosity to further mimic the properties of a human bone [18]. Various methods to produce porous bioceramics have been investigated, including polymer replication [19,20], solid freeform fabrication [21], rapid prototyping [22,23,24], and freeze-casting [25,26,27]. Among these, freeze-casting is a simple and effective fabrication procedure to obtain customized porous structures and has been applied in numerous studies [28,29].

Although porous ceramics are good for biomedical applications, the demand for hierarchical porous structures, also known as functionally graded structures, is increasing. By mimicking the hierarchical structure of a real bone, these hierarchical porous structures are expected to reduce the stress-shielding effect [30,31,32,33]. Furthermore, adopting functionally graded structures in porous ceramic structures has the advantage of combining parts with different porosities. Regions with a high porosity facilitate the transport of bodily fluids, which enhances bone regeneration. On the other hand, less-porous regions support the mechanical load as load-bearing parts, which are essential for bone grafts [26,34].

Herein, we propose a simple and efficient fabrication method for porous HA scaffolds with graded porosity using sequential freeze-casting. HA scaffolds with homogeneous porous structures were fabricated by freeze-casting with different powder concentrations to control the porosity and pore size. Sequential freeze-casting utilizing slurries with different HA vol % slurries was adopted to achieve functionally graded structures. The crystalline phases of the scaffolds were checked by X-ray diffraction (XRD) analysis. Scanning electron microscopy (SEM) and micro computed tomography (micro-CT) were used to observe the resulting structures, and the compressive strengths were measured by compression testing. The compressive strengths of the scaffolds with various structures were examined, and in vitro cell attachment and proliferation tests were conducted to demonstrate biocompatibility.

## 2. Experimental Section

### 2.1. Materials and Manufacturing

**Figure 1:**
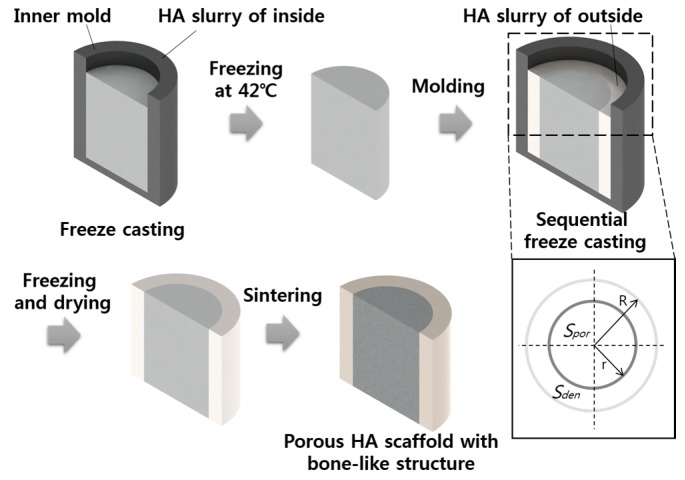
Schematic of the sequential freeze-casting process and the design of the bone-like structure (HA-hydroxyapatite; *Spor*-surface area of porous region; *Sden*-surface area of dense region; R-radius of the larger mold; r-radius of the smaller mold).

HA powder (Ca_10_(PO_4_)_6_(OH)_2_; Alfa Aesar Co., Milwaukee, WI, USA) and camphene (C_10_H_16_; Sigma Aldrich, St. Louis, MO, USA) were used as the starting materials. HA/camphene slurries with different HA contents (10, 15, 20, 25, 40, and 50 vol %) were prepared by ball milling at 60 °C, with the addition of dispersant (Hypermer KD-4; UniQema, Everberg, Belgium). The generated slurries were poured into a 20-mm cylindrical mold, and the solidifying process was conducted at 42 °C. For sequential freeze-casting, the molds were produced with the sizes shown in Figure 1; the solidifying process of the first HA slurry with low HA contents was conducted in a mold with a smaller radius *r*; furthermore, the solidified green body was removed from the inner mold and located in a larger mold with a radius *R*, with the residual vacant space filled with slurries with high HA contents. After solidification, the green body with a graded concentration of HA was freeze dried to sublimate camphene and generate pores, and sintering was conducted at 1250 °C for 2 h. For further applications, inversely structured scaffolds were fabricated using the same sequence by switching the HA contents of the starting slurries between the inner and outer parts.

### 2.2. Characterization

#### 2.2.1. Porous Structure

To characterize the crystalline phases, XRD (D8-Advance; Bruker Co., Karlsruhe, Germany) analysis was conducted for the scaffolds with different vol % of HA. The phases were analyzed in the 2 theta region of 20–50°, with a scanning rate of 1°/min. The pore structures were characterized by SEM (JSM-6360; JEOL Techniques, Tokyo, Japan), and the porosity and pore size were further investigated using micro-CT (Skyscan 1173 X-ray m-tomography System, Bruker microCT, Kontich, Belgium) with the following parameters: 1.0-mm aluminum filter, 0.2° rotation step, 180° rotation, average of five frames, five random movements, voltage of 100 kV, and current of 80 μA. Porosity and pore size were analyzed from the obtained images using CTAn (Bruker, Kontich, Belgium) analysis software. Three-dimensionally constructed images were acquired using CTVox (Bruker, Kontich, Belgium) software.

#### 2.2.2. Compressive Strength Measurement

The compressive strengths of the fabricated graded porous HA scaffolds were measured using an Instron 5582 System (Instron, Norwood, MA, USA) under a displacement-controlled mode, with a cross-head speed of 1 mm/min. The five specimens used to examine the compressive strengths were 20 mm in diameter and 20 mm in height. 

#### 2.2.3. Biocompatibility

In vitro cellular responses were examined using MC3T3-E1 preosteoblasts. Cells were cultured in a humidified incubator with 5% CO_2_ at 37 °C. The culturing medium comprised alpha-minimum essential medium (Welgene Co., Ltd., Gyeongsan, Korea), 10% fetal bovine serum (FBS), and 1% penicillin streptomycin. Initial cell attachment was checked after 5 h of culturing using confocal laser scanning microscopy (CLSM; LSM 510 NLO, Carl Zeiss, Oberkochen, Germany). The cells which were attached on the scaffolds were fixed with 4% paraformaldehyde, and disrupted and blocked by 0.1% of triton X-100 and 1% of bovine serum albumin. Alexa Fluor 546 phalloidin (Molecular Probes, Eugene, OR, USA) and ProLong Gold antifade reagent with 4′,6-diamidino-2-phenylindole (Molecular Probes, Eugene, OR, USA) were used to stain the cytoplasms and nuclei of the cells, respectively. Cell proliferation on the porous HA scaffolds with bone-like structures was determined after three and five days of culturing using the MTS (methoxyphenyl tetrazolium salt) proliferation assay technique and was then compared, with a tissue culture plate (TCP) as the control specimen. After culturing, the specimens were rinsed twice with Dulbecco’s phosphate buffered saline. The rinsed specimens were immersed into the FBS-absent culturing medium mixed with a 10% cell proliferation assay kit solution (CellTiter 96 AQueous One Solution Cell Proliferation Assay). The absorbance of the solution was checked at 490 nm using a microplate reader (Model 550; Biorad, Seoul, Korea), after 2 h of culturing.

## 3. Results and Discussion

### 3.1. Crystalline Structure

**Figure 2:**
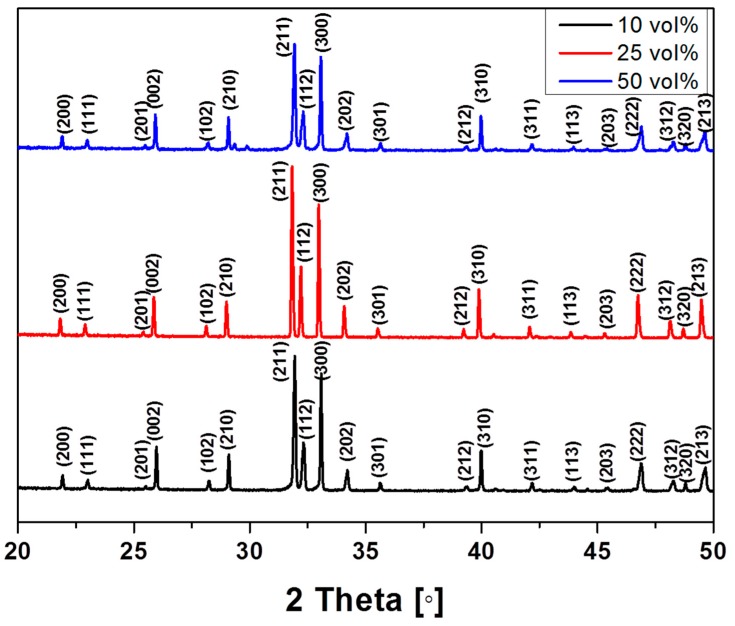
X-ray diffraction (XRD) pattern of porous HA scaffolds with various initial HA contents.

The crystalline structures of the porous HA scaffolds with different powder concentrations (10, 25, and 50 vol %) were analyzed by XRD. The representative XRD pattern of HA was observed, even after sintering at 1250 °C (Figure 2). The XRD patterns of the produced scaffolds only show peaks related to HA, indicating that no contamination occurred during freeze drying. The XRD patterns of the scaffolds containing HA contents of 15, 20, and 40 vol % were the same as those shown in Figure 2 (data not shown), indicating that varying the HA content in the scaffolds did not affect the crystalline phase of HA itself.

### 3.2. Porous Structures of Homogeneous Porous HA Scaffolds

**Figure 3:**
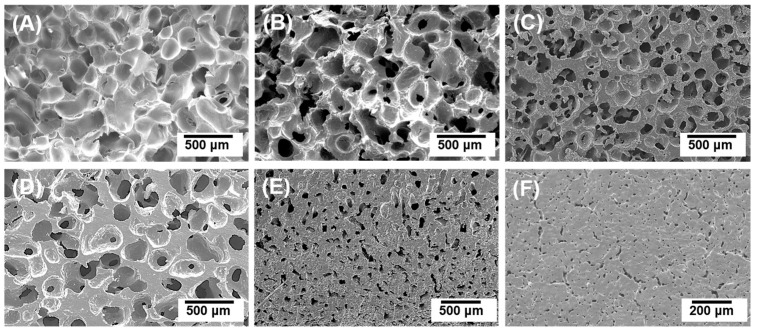
Typical scanning electron microscopy (SEM) images of porous HA scaffolds with initial HA contents of (**A**) 10 vol %; (**B**) 15 vol %; (**C**) 20 vol %; (**D**) 25 vol %; (**E**) 40 vol %; and (**F**) 50 vol %.

Six types of porous HA scaffolds were fabricated with different initial HA powder concentrations (10, 15, 20, 25, 40, and 50 vol %). As shown in Figure 3, all the scaffolds were successfully fabricated without any noticeable cracks or defects. Their structures were comparable to the microstructure of real bone composed of trabecular bone (Figure 3A–D) and cortical bone (Figure 3E,F) [35,36,37].

**Figure 4:**
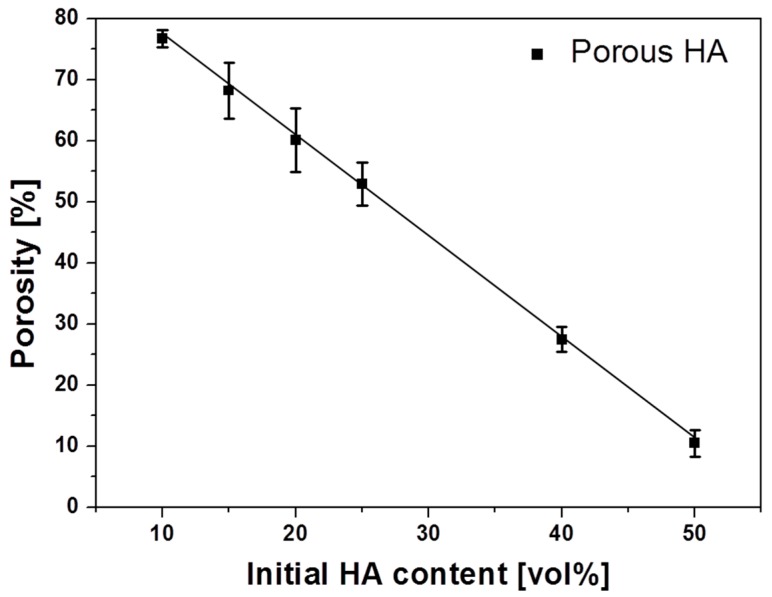
Porosity of porous HA scaffolds as a function of initial HA content.

As the HA powder concentration increased from 10 to 50 vol %, the porosity decreased from 78% to 10%. These values are within the range of porosities reported in several studies that used the freeze-casting method [25,27,38]. In particular, the scaffolds containing 45 and 50 vol % HA, which showed dramatic decreases in porosity and pore size, were examined. Producing scaffolds with HA contents exceeding 50 vol % was challenging because of the high viscosity of the HA slurry. Utilizing the calculated porosity of each scaffold, the relationship between the initial HA content (*v*) and scaffold porosity (*p*) was theoretically determined as a negatively proportional linear profile: p=93.3−1.65v (Figure 4). Using this equation, HA scaffolds with the desired porosity can be easily fabricated with a simple calculation.

### 3.3. Materials and Manufacturing

**Figure 5:**
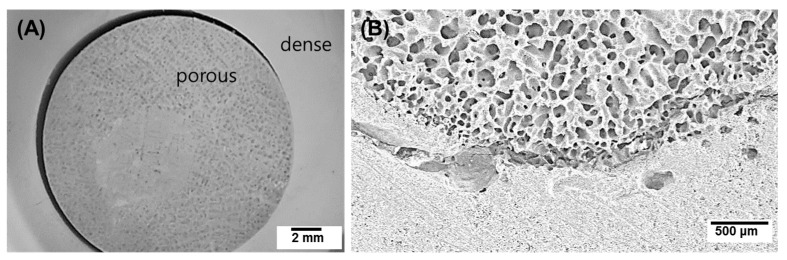
(**A**) Optical and (**B**) SEM images of a porous HA scaffold with a bone-like structure: inner initial HA content = 10 vol %, and outer initial HA content = 50 vol %.

Figure 5 shows the bone-like structure of a porous HA scaffold formed by sequential freeze-casting with two different initial HA concentrations: 10 vol % for the inner part and 50 vol % for the outer part. The optical image shows a significant difference between the inner and outer layers with respect to the generated pores (Figure 5A). Although there was tight adhesion between the two layers as a green body, delamination occurred because of the different shrinkage behavior in the two layers during sintering [39].

**Figure 6:**
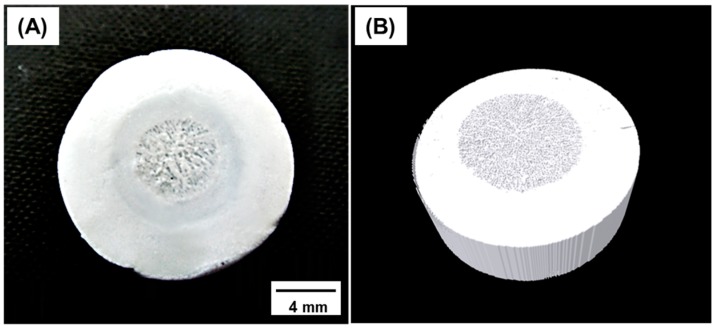
(**A**) Optical and (**B**) reconstructed micro-CT images of a porous hydroxyapatite (HA) scaffold with a bone-like structure and inner and outer initial HA contents of 15 and 40 vol %, respectively.

As shown in Figure 6, we successfully fabricated a scaffold with different initial HA concentrations in the inner (15 vol %) and outer (40 vol %) parts. The structural characteristics of the two parts were confirmed by optical microscopy, and the internal structure was further investigated by micro-CT (Figure 6). During sequential freeze-casting, the solidified HA slurry of the outer part adhered to the readily fabricated inner green body, without any interferences. Because of the sufficient adhesive property, the interface maintained its structure, even after sublimation and sintering. The final structure was hierarchical, with two regions of different porosities, resembling the structure of a bone [35,36,37].

**Figure 7:**
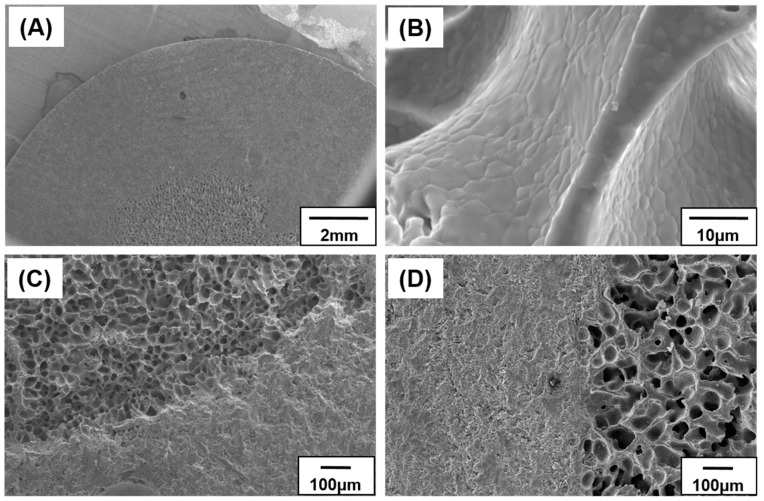
SEM images of a porous hydroxyapatite (HA) scaffold with bone-like structure: (**A**) low-magnification; (**B**) high-magnification; (**C**) cross section perpendicular to the axial direction; and (**D**) cross section parallel to the axial direction.

Figure 7A shows the overall microstructures of the porous and dense parts of the scaffold. The two parts demonstrated noticeably different structures and pore characteristics without anisotropy. No cracks or micro-pores were found in the pore walls after sintering (Figure 7B). This implies that the HA particles were efficiently sintered, regardless of the micro-pores created during the sublimation of camphene. The presence of micro-pores within the HA walls can be detrimental to the mechanical properties of bone grafts; however, the microstructure of the produced dense HA wall showed no noticeable pores. The cross-sectional structures perpendicular and parallel to the axial direction are shown in Figure 7C,D, respectively. As illustrated in both the images, a functionally graded porous structure was formed, as initially designed. Moreover, the interface between the two regions remained intact during the solidification step.

The porosities and pore sizes of scaffolds with different *S_den_*/*S_por_* (ratio of the surface area of the relatively dense part (*S_den_*) to that of the porous part (*S_por_*)) were examined by micro-CT (Table 1). The pore characteristics of the inner and outer parts of each scaffold were nearly identical because the HA contents were fixed to 15 and 40 vol %, respectively. The increase in the proportion of the dense part resulted in a decrease in the overall porosity. There was no significant difference between the measured porosity values analyzed by micro-CT and those theoretically calculated from the porosity and area.

**Figure 8:**
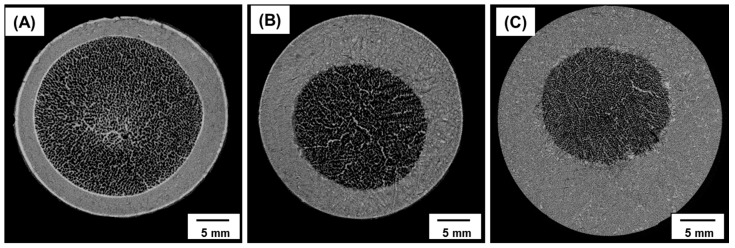
Micro-CT images of porous HA scaffolds with bone-like structures: (**A**) *S_den_*/*S_por_* = 0.44; (**B**) *S_den_*/*S_por_* = 1.25; and (**C**) *S_den_*/*S_por_* = 3 (*Sden*-surface area of dense region; *Spor*-surface area of porous region).

The morphologies of the internal pores with various ratios of dense to porous parts were investigated based on micro-CT images (Figure 8). Although the interface between the two parts was clearly detected in each image, no detachments were observed. The observed double-layered structures resemble the structure of real bone, as indicated above in Section 3.3. From the micro-CT analysis, the interconnectivity of the porous regions was greater than 99% due to the dendritic growth of camphene, which was sublimated to generate open pores [40,41]. Considering the difficulties in creating a layered structure without defects, the proposed sequential freeze-casting method is a promising technique for several applications [40].

### 3.4. Compressive Strengths of Porous HA Scaffolds with Bone-Like Structures

**Figure 9:**
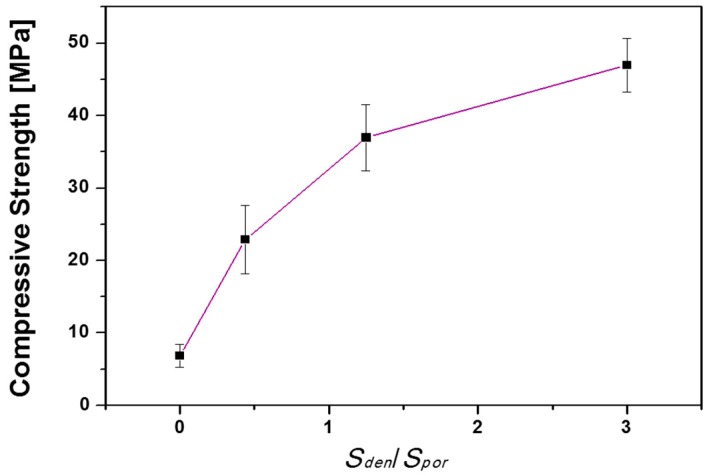
Compressive strength of porous HA scaffolds with bone-like structures as a function of *S_den_*/*S_por_*.

The compressive strengths of the scaffolds with different ratios of the dense part to the porous part were evaluated (Figure 9). The compressive strength increased from 7 to 47 MPa when *S_den_*/*S_por_* increased from 0 to 3. The enhanced compressive strength was mainly attributed to the increase in the portion of the dense part. By controlling the *S_den_*/*S_por_* ratios of the scaffolds, the structural and compressive features can be customized to match those of the surrounding bones at the implant site [18,42]. The compressive strengths of fabricated porous HA scaffolds with bone-like structures were within the range of 20–50 MPa, which are in the range of those in tibia, femur, and trabecular bone, with or without marrow [42,43].

### 3.5. Biocompatibility Evaluation

**Figure 10:**
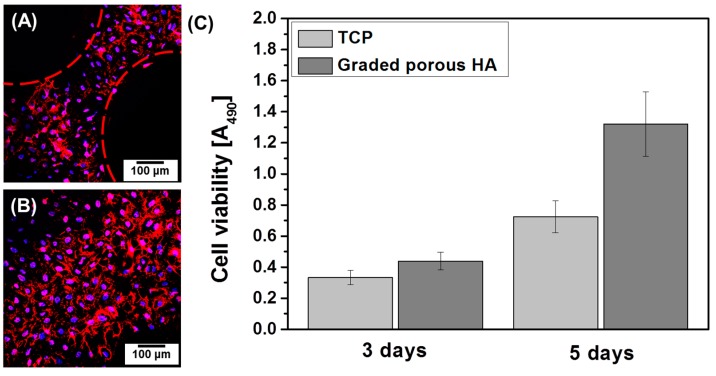
CLSM images of preosteoblast cells cultured for 5 h on porous HA scaffolds with bone-like structures: (**A**) porous part and (**B**) dense part; (**C**) Cell viabilities of the preosteoblast cells cultured for three and five days on porous HA scaffolds with bone-like structures.

Initial preosteoblast cell attachment on the porous HA scaffolds with bone-like structures was confirmed by CLSM. As shown in Figure 10A,B, preosteoblast cells were well attached to both the dense and porous parts of the porous HA scaffolds, as confirmed by their multiple nuclei (stained in blue) and spread cytoplasm (stained in red), even after a short period of culturing (5 h). In particular, excellent biocompatibility was demonstrated by the spread cytoplasm and stretched filopodia [44]. However, the images only focused on cells on the surface; the cells in the pores (within the dashed red lines) were not observed. The cell proliferation properties were evaluated after three and five days of culturing on a TCP (used as a control) and the fabricated porous HA scaffolds with bone-like structures as shown in Figure 10C. After three days of culturing, the cell proliferation rate was slightly higher in the porous HA scaffolds than in the control, although the difference was not significant. However, a significant difference in proliferation was observed when the culturing time was increased to five days. Because of the outstanding biocompatibility of HA, excellent initial cell attachment and cell proliferation were obtained.

### 3.6. Tailored Application of the Sequential Freeze-Casting Method

**Figure 11:**
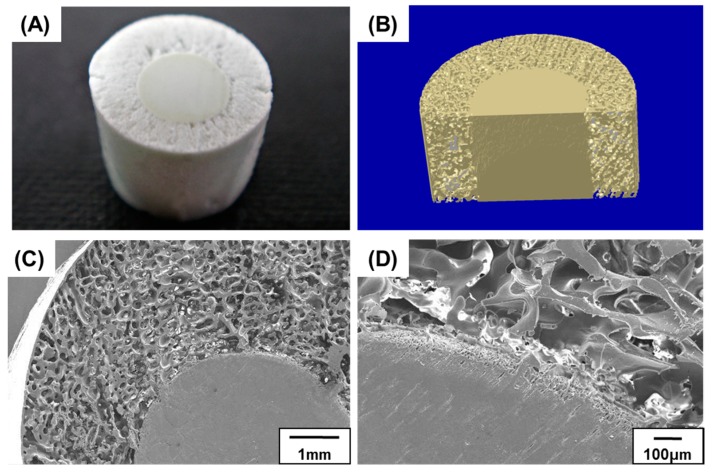
(**A**) Optical; (**B**) micro-CT; and (**C**,**D**) SEM images of a porous hydroxyapatite (HA) scaffold possessing a dense internal core and porous outer layer.

Inversely structured scaffolds consisting of highly porous outer parts and dense inner parts were successfully obtained by sequential freeze-casting (Figure 11). While the outer porous part can enhance interlocking with the surrounding bone under physiological conditions, the inner dense part acts as a load-bearing region because of its superior compressive strengths. Accordingly, scaffolds with this structure would be useful for implanting at sites wherein early bone-to-implant contact is required. By tailoring the ratio between the two parts and their structural organization, the functional diversity of graded porous HA scaffolds can be expanded [45,46].

## 4. Conclusions

Porous HA scaffolds with different HA contents were successfully fabricated using a freeze-casting technique. Regardless of the HA content of the scaffold, the phase of each scaffold was identical, and no non-HA phases were detected. However, the structures of the scaffolds with different HA contents were significantly different. As the HA content increased, the porosity and pore size decreased proportionally.

To mimic the natural bone structure, porous HA scaffolds with bone-like structures were produced by sequential freeze-casting. Despite the two-step freeze-casting process, the interface between the inner and outer parts was well-connected, even after solidification, sublimation, and sintering. As *S_den_*/*S_por_* increased, the overall porosity decreased and the compressive strengths were enhanced. Consequently, the mechanical and structural characteristics could be adjusted while maintaining the bone-like structure, by altering the proportions of the porous and dense parts. In vitro cell attachment and proliferation tests using preosteoblast cells confirmed the biocompatibility of the scaffolds. Preosteoblast cells were well spread on the scaffolds, and the cell viability increased significantly after three and five days of culturing.

Utilizing a sequential freeze-casting method, inversely structured scaffolds with dense inner parts and porous outer parts were generated without any difficulties. These structures can rapidly interlock with the surrounding tissue and bone because of the large pores on the outer surface. The dense part also provides load-bearing support, which compensates for the lowered compressive strengths of the highly porous part.

## Figures and Tables

**Table 1:** Pore characteristics of porous HA scaffolds with bone-like structures.

Name	*R* [mm]	*r* [mm]	*S_den_*/*S_por_*	Porosity [%]			Pore size [μm]	
				Inner	Outer	Overall	Inner	Outer
B0	-	20	0	68.2 ± 3.5	-	68.2 ± 3.5	150 ± 33	-
B1	20	16.7	0.44	66.5 ± 4.1	27.4 ± 2.1	55.7 ± 5.5	145 ± 23	41 ± 11
B2	20	13.3	1.25	67.2 ± 2.5	28.2 ± 3.6	45.6 ± 4.1	148 ± 37	42 ± 14
B3	20	10	3	66.5 ± 3.7	26.5 ± 2.3	37.7 ± 5.2	161 ± 21	46 ± 9

*Sden*-surface area of dense region; *Spor*-surface area of porous region; R-radius of the larger mold; r-radius of the smaller mold.
